# Hereditary angioedema diagnosis: Reflecting on the past, envisioning the future^☆^

**DOI:** 10.1016/j.waojou.2025.101060

**Published:** 2025-05-14

**Authors:** Anete S. Grumach, Marc A. Riedl, Lei Cheng, Siddharth Jain, Daniel Nova Estepan, Andrea Zanichelli

**Affiliations:** a Faculdade de Medicina, Centro Universitario Faculdade de Medicina ABC (CEUFMABC), Santo André, Brazil; b Division of Allergy and Immunology, Department of Medicine, University of California San Diego, La Jolla, CA, USA; c Department of Otorhinolaryngology & Clinical Allergy Center, The First Affiliated Hospital, Nanjing Medical University, Nanjing, China; d International Centre for Allergy Research, Nanjing Medical University, Nanjing, China; e Takeda Development Center Americas, Inc., Lexington, MA, USA; f Operative Unit of Medicine, Angioedema Center, IRCCS Policlinico San Donato, San Donato Milanese, Milan, Italy; g Dipartimento di Scienze Biomediche per la Salute, University of Milan, Milan, Italy

**Keywords:** C1 inhibitor, C4, Diagnosis, Genetic testing, Hereditary angioedema

## Abstract

Individuals with hereditary angioedema (HAE), a rare disease most frequently associated with deficiency (HAE-C1INH-Type1) or dysfunction (HAE-C1INH-Type2) of C1 inhibitor (C1INH), continue to experience frequent misdiagnoses and long delays in diagnosis, preventing appropriate management strategies and placing the patients at continued risk of inappropriate management of painful, debilitating, and potentially fatal swelling attacks. Physician education to increase HAE awareness is important to initiate diagnostic testing for patients who may be at risk of HAE. Standard tests for diagnosing HAE-C1INH-Type1 and HAE-C1INH-Type2 include measurements of antigenic C4 level, antigenic C1INH level, and C1INH function; in contrast, known subtypes of HAE due to normal C1INH can only be confirmed through genetic testing. Current diagnostic tests have certain limitations related to sample handling, storage, and transportation; concerns about the sensitivity and specificity of current assays have also been reported. Furthermore, the accessibility of diagnostic testing for HAE is not universal. Therefore, there is a persistent need for robust and accessible diagnostic tools for HAE. In this review, we provide an overview of currently available assays for HAE diagnosis and summarize some of the novel diagnostic tools that may aid in overcoming diagnostic challenges in HAE and supporting the care of patients with HAE.

## Introduction

Hereditary angioedema (HAE) is a rare genetic disease with different recognized, genetically identifiable forms.^1^ Both HAE due to C1 inhibitor (C1INH) deficiency (HAE-C1INH) type 1 (HAE-C1INH-Type1) and type 2 (HAE-C1INH-Type2) are associated with mutations in the *SERPING1* gene coding for C1INH.^1^ HAE-C1INH-Type1 (approximately 80–91% of HAE cases) is caused by a quantitative deficiency in C1INH protein,^2^, ^3^, ^4^ and HAE-C1INH-Type2 (approximately 9–15% of HAE cases) is caused by a dysfunction in C1INH protein.^2^^,^^3^ HAE due to normal C1INH (HAE-nC1INH) is a group of very rare disorders of recurrent angioedema due to hereditary causes; patients with HAE-nC1INH present with normal levels of antigenic C1INH and functional C1INH (fC1INH).^1^^,^^3^ Although the exact prevalence of HAE-C1INH remains difficult to verify, HAE-nC1INH is expected to be at least several times less frequent than HAE-C1INH.^5^, ^6^, ^7^ HAE-nC1INH can be classified based on the causative genes; mutations in blood clotting factor XII (*F12*), angiopoietin-1 *(ANGPT-1*), plasminogen (*PLG*), kininogen 1 (*KNG1*), myoferlin (*MYOF*), and heparan sulfate 3-*O*-sulfotransferase 6 (*HS3ST6*) genes are known to be associated with HAE-nC1INH.^1^ Recent reports also included mutations in carboxypeptidase N (*CPN1*) and disabled homolog 2-interacting protein (*DAB2IP*) genes that may be associated with HAE-nC1INH.^1^^,^^8^^,^^9^ Causative genes remain unknown in some patients with HAE-nC1INH and will continue to be explored in the future.^10^

Clinical manifestations of HAE include recurrent swelling that may involve skin and subcutaneous tissues and/or abdominal organs.^1^ Although all HAE attacks can be painful, disfiguring, and debilitating, laryngeal attacks are particularly dangerous as they may cause death by asphyxiation if untreated.^11^

Patients with HAE face challenges in receiving correct diagnosis, a feature common to rare diseases overall. Approximately 6000–8000 rare diseases have been identified to date.^12^ People living with rare diseases often experience lengthy delays in achieving correct diagnosis, which can stem from lack of awareness both from the physicians and the patients themselves, especially if the rare disease shares clinical features with more common conditions.^12^, ^13^, ^14^ Support from the patient communities and support groups may help to raise awareness of the rare diseases and, in turn, shorten the diagnostic journeys.^15^ Among the difficulties in HAE diagnosis, many physicians report misdiagnosis as the top diagnostic challenge.^16^^,^^17^ This is unsurprising, considering the general lack of HAE awareness in clinical practice and the fact that many of the HAE signs and symptoms are nonspecific and are often shared with other forms of angioedema.^18^^,^^19^ Long diagnostic delays from HAE symptom onset to HAE diagnosis have also been reported.^20^ Up to 90% of HAE deaths due to asphyxiation occur in patients that are undiagnosed; therefore, reducing diagnostic delays in HAE is important to reduce mortality and morbidity associated with HAE.^21^^,^^22^

In this narrative review, we provide an overview of the current diagnostic algorithm for HAE diagnosis and describe novel and in-development methods for diagnosing HAE.

## Current HAE diagnostic algorithm

### Patient history and clinical presentation

Physician awareness of HAE clinical history is the essential first step to HAE diagnosis, especially in patients with a family history of HAE. Because patients with HAE may present with symptoms affecting different parts of the body, they may be seeing physicians of different specialties for their symptoms; these may include, but are not limited to, primary care physicians, pediatricians, allergologists/immunologists, dermatologists, gastroenterologists, and/or emergency department physicians.^18^ Unsurprisingly, physician awareness of HAE varies among physicians of different specialties.^19^^,^^23^ The specific considerations required to effectively diagnose and treat HAE for physicians by specialty have been summarized in a review by Magerl et al (2023).^18^

Clinical suspicion of HAE should be raised in patients presenting with a history of recurrent swelling of the skin, abdominal pain, and/or laryngeal edema, with further suspicion being raised when patients have a positive family history of HAE; onset in childhood or adolescence; lack of response to antihistamines, glucocorticoids, omalizumab, or epinephrine; prodromal signs or symptoms; and/or absence of urticaria.^1^ However, urticaria has been described in the rare forms of HAE-nC1INH with *CPN1* and *DAB2IP* mutations.^8^^,^^9^ As HAE is an inherited disorder, family member screening for HAE is recommended once HAE diagnosis is confirmed in a patient, although approximately 25% of patients with HAE-C1INH may present with de novo mutations.^1^^,^^24^

### Complement testing

**Fig. 1:**
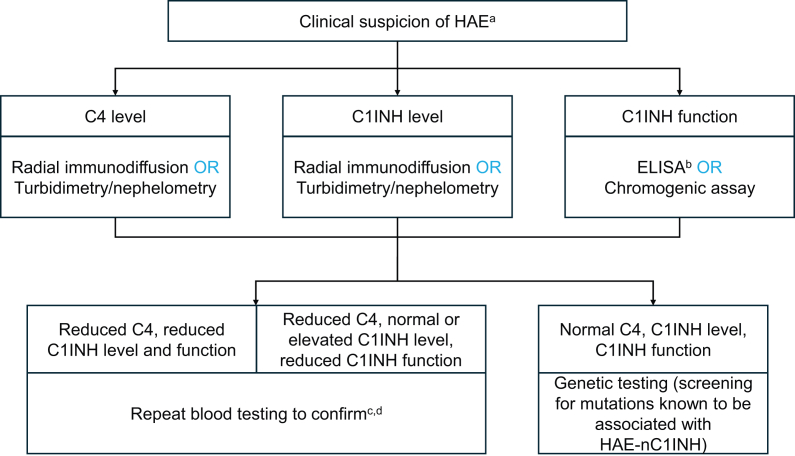
Summary of current diagnostic algorithm for HAE.^1^^,^^26^ C1INH = C1 inhibitor; ELISA = enzyme-linked immunosorbent assay; HAE = hereditary angioedema; HAE-nC1INH = hereditary angioedema due to normal C1 inhibitor. ^a^Patient presents with a history of recurrent skin swelling or gastrointestinal attacks (abdominal pain and/or laryngeal edema), especially in patients with family history; onset in childhood or adolescence; recurrent and painful abdominal symptoms; upper airway edema; lack of response to antihistamines, glucocorticoids, omalizumab, or epinephrine; presence of prodromal signs; and/or the absence of urticaria. ^b^Based on C1INH–C1s protease complex formation. ^c^In patients with no family history and symptom onset at the age of >30 years, C1q levels should also be assessed to exclude acquired angioedema due to C1INH deficiency. ^d^Access to effective on-demand treatment should not be delayed if initial laboratory testing supports the diagnosis of HAE when this condition is clinically suspected

To establish HAE diagnosis, the international guidelines for HAE management recommend the testing of blood levels of antigenic C4, antigenic C1INH, and fC1INH to be performed in all patients with suspected HAE. Furthermore, it is suggested that this testing is repeated in patients who test positive for HAE for the confirmation of diagnosis, particularly in cases where the fC1INH results are ambiguous.^1^ However, it is important not to delay access to effective on-demand treatment if initial laboratory testing supports the diagnosis of HAE when this condition is clinically suspected.^1^ Antigenic C4 is usually reduced in both patients with HAE-C1INH-Type1 and HAE-C1INH-Type2; however, some patients with HAE-C1INH-Type1 or HAE-C1INH-Type2 may present with normal antigenic C4 levels between attacks.^1^^,^^25^ Both antigenic C1INH level and C1INH function are decreased in patients with HAE-C1INH-Type1, whereas patients with HAE-C1INH-Type2 have normal or elevated antigenic C1INH level but reduced C1INH function;^1^ therefore, fC1INH is essential for the diagnosis of HAE-C1INH-Type1 and HAE-C1INH-Type2.^1^ In patients with laboratory findings suggestive of HAE-C1INH-Type1 (reduced antigenic C1INH and antigenic C4 levels as well as C1INH function) without family history and late age of onset (>30 years), diagnosis of acquired angioedema due to C1INH deficiency should be excluded; measurement of C1q (typically normal in HAE-C1INH-Type1 and reduced in approximately 75% of cases of acquired angioedema due to C1INH deficiency) may be helpful for differential diagnosis.^1^ The current diagnostic algorithm for HAE is summarized in Fig. 1.

The levels of antigenic C1INH and antigenic C4 can be assessed using routine laboratory techniques, including radial immunodiffusion and turbidimetry/nephelometry assays.^26^ The standard laboratory methods to evaluate fC1INH include enzyme-linked immunosorbent assay (ELISA) and chromogenic assay. The fC1INH ELISA is based on C1s protease inhibition and measures the C1INH function via the detection of complexes formed following C1 activation.^27^^,^^28^ The fC1INH chromogenic assay measures the C1INH function via the residual C1s protease activity following the introduction of C1s protease–specific substrate; substrate cleavage forms a colored product, and reduced color intensity reflects C1s protease inhibition by C1INH.^27^^,^^29^ Overall, both fC1INH ELISA and fC1INH chromogenic assays have been reported to provide similar results,^27^^,^^29^^,^^30^ although some studies have raised concerns with the specificity of fC1INH chromogenic assay^30^ and the sensitivity of fC1INH ELISA.^31^

Both fC1INH ELISA and fC1INH chromogenic assays require correct sample handling as well as low-temperature conditions (refrigeration or freezing) for storage and shipping to avoid sample decay and incorrect results.^30^^,^^32^ For example, in one study, suboptimal sample handling (interlaboratory shipping without temperature control) has been associated with incorrect results of fC1INH chromogenic assay (falsely pathological results in healthy subjects).^32^ Furthermore, fC1INH ELISA may yield equivocal results,^27^^,^^28^ which do not definitively confirm or reject HAE diagnosis via reduced or normal fC1INH level, respectively, necessitating repeated testing.^27^ This suggests a persistent need for reliable assays for HAE diagnosis, which may help to simplify the diagnostic algorithm for HAE.

### Genetic testing

A supplementary method for diagnosis of HAE-C1INH-Type1/HAE-C1INH-Type2 is genetic testing, although it is overshadowed by effective biochemical tests (antigenic C4, antigenic C1INH level, fC1INH) where access to these tests is available.^1^^,^^33^ Sequencing of the *SERPING1* gene coding for C1INH may provide supporting evidence for HAE diagnosis in specific cases. In particular, *SERPING1* genotyping may be helpful for prenatal diagnosis or diagnosis of children aged <1 year if the gene variant in the parent with HAE is known; as a method for family screening in suspected de novo cases of HAE; or as confirmation of HAE diagnosis when biochemical assay results are inconclusive.^1^^,^^24^^,^^33^ Furthermore, targeted Sanger sequencing may be a preferrable method for family screening once an index case is established in some developing countries, providing a cost-effective alternative to biochemical testing.^34^ However, some studies showed that *SERPING1* sequencing may have low sensitivity (ie, failure to correctly identify patients with biochemically proven C1INH deficiency).^35^ When next-generation sequencing is used, certain mutations, such as large deletions in *SERPING1*, may be missed with the whole exome sequencing. The detection of these mutations may require more costly whole genome sequencing, suggesting that the sensitivity may vary dependent on the specific sequencing technique applied.^36^

In HAE-nC1INH, antigenic C4, antigenic C1INH, and fC1INH levels are normal; existing biochemical tests for these analytes may be used to rule out a diagnosis of HAE-C1INH when patients meet other criteria for diagnosis.^1^ It has also been suggested to confirm the lack of response to second-generation antihistamines and mast cell–targeted therapies in patients with clinical suspicion of HAE and normal antigenic C4, antigenic C1INH, and fC1INH levels.^37^ In patients with HAE-nC1INH caused by known mutations, genetic testing is currently the only laboratory method able to provide a confirmatory diagnosis.^1^ However, since genetic testing is limited to screening for currently identified mutations, it poses a pronounced obstacle to ascertaining the diagnosis, as a large proportion of patients with HAE-nC1INH may have an unknown mutation.^1^^,^^24^ As the causative gene is often unknown in these patients, and access to laboratories that provide genetic sequencing for variants associated with HAE-nC1INH is not universal, the unmet need for diagnostic biomarker-based assays remains notable (see Jones et al., 2023 for a detailed summary of challenges in HAE-nC1INH diagnosis).^38^^,^^39^

## Access to diagnostic tools

The rates of HAE diagnosis and the delay between HAE symptom onset to diagnosis vary in different countries and regions,^20^^,^^40^, ^41^, ^42^, ^43^, ^44^, ^45^ which is at least in part due to variability in awareness of HAE and access to diagnostic tests. Limited access to diagnostic tests has been reported in certain regions of the world (eg, India and other countries from the Asia Pacific region).^34^^,^^35^^,^^46^ Unsurprisingly, it has been estimated that more than 95% of patients with HAE from these countries may remain undiagnosed.^34^^,^^35^

Limited access to comprehensive complement testing in some regions of the world may further complicate the diagnostic algorithm for HAE. For example, in Brazil and countries in the Asia Pacific region, C4 may be the only widely available complement test for HAE diagnosis, with antigenic C1INH and/or fC1INH testing available only in the specialized laboratories or reference centers.^35^^,^^40^^,^^47^ Furthermore, antigenic C1INH and/or fC1INH testing may be cost prohibitive in some countries and regions; for example, antigenic C1INH testing is not covered by the national health insurance system in Japan.^40^ This may lead to physician reluctance to pursue specific tests. As C4 levels may be normal in patients with HAE,^25^ the reliance on only C4 for screening may cause false-negative results or require repeated C4 testing, potentially extending the time to definitive diagnosis.^34^ In some countries (eg, India and China), access to fC1INH testing is very limited, posing a specific problem for diagnosis of HAE-C1INH-Type2, which is characterized by normal levels of antigenic C1INH, with potentially normal C4 levels as well.^1^^,^^35^^,^^40^ Even if fC1INH testing is available in reference centers, resource-constrained countries may face challenges owing to the sample handling requirements of the standard fC1INH tests, as described in detail below.^34^^,^^40^^,^^47^ The complexity of existing diagnostic algorithms highlights the need for better access to diagnostic testing (especially fC1INH) for HAE.

In regions with limited access to specialist healthcare providers, regional referral pathways may contribute to improved diagnosis, management, and outcomes.^48^ One of the initiatives to raise HAE awareness and provide excellence in angioedema management is the Angioedema Center of Reference and Excellence program, launched by the Global Allergy and Asthma European Network and HAE International.^49^ Patient advocacy groups such as HAE International also contribute to raising the awareness of HAE. As an example of furthering the reach of reference centers, Hong Kong–Macau Severe Hives and Angioedema Referral Pathway has been created to promote multidisciplinary collaboration in urticaria and angioedema management in the related Great Bay of China region.^48^ Similarly, the Zhejiang Province HAE Specialty Diagnosis and Treatment Collaborative Network was established to enable multidisciplinary collaboration on the HAE diagnosis and treatment in the Zhejiang province of mainland China.^50^ In other cases, patient samples may be collected locally and sent for analysis in a central laboratory — this was suggested as one of the solutions to allow for more widespread testing in locations with limited capabilities for laboratory testing (eg, underserved geographical regions and rural locations).^51^ A similar approach has been reported in a survey of Canadian laboratories.^52^ The results of this survey showed that only a small number of surveyed Canadian laboratories had the capabilities to perform antigenic C1INH level and C1INH function testing, with the rest of the laboratories outsourcing these tests to other Canadian and US laboratories.^52^ Although this system increased the physician accessibility of HAE diagnostic assays, many physicians reported low confidence in the results of fC1INH assays due to concerns about handling, shipping, and storing of samples.^52^ These findings further highlight the importance of novel diagnostic methods that would enable analysis in central laboratories via improved sample storage, handling, and transportation. Dried blood spot (DBS)–based assays that have easy sampling methodology and allow sample storage and transportation at ambient temperatures have a potential to partially contribute to the wider adoption of centralized HAE testing and, in turn, to improved accessibility of diagnostic testing for HAE.

## New and in-development diagnostic tools for HAE

### DBS assays

To address the limitations of existing HAE diagnostic assays, new diagnostic methods continue to be developed. DBS-based assays are garnering increasing interest in various diagnostic fields, including testing for HAE.^53^, ^54^, ^55^ The advantages of DBS-based assays include sampling from capillary blood, which is less invasive and less complicated compared with sampling from venous blood, as well as simplified sample transportation at ambient conditions due to increased stability.^51^^,^^54^^,^^56^^,^^57^ One of the novel DBS-based assays for HAE diagnosis measures fC1INH from DBS samples using mass spectrometric methods.^54^ For this assay, C1s protease substrate is introduced after incubation of DBS samples with excess C1s protease; the product of the enzymatic reaction between C1s protease and its substrate is measured by liquid chromatography tandem mass spectrometry.^54^ The same group also reported a multiplexed DBS-based assay to quantify antigenic C1INH level, antigenic C4, and C1q from DBS samples.^53^ For this multiplexed assay, the blood proteins are extracted from DBS samples and digested with trypsin. Following enzymatic digestion, signature peptides derived from C1INH, C4, and C1q proteins are quantified using liquid chromatography tandem mass spectrometry.^53^ Another group reported a DBS-based assay for C1INH and C4 protein quantification using a different mass spectrometric method, combined with genetic confirmation using DNA extracted from the DBS samples.^55^ For the C1INH and C4 protein quantification, proteins are extracted from the DBS samples, followed by disulfide bridge reduction, alkylation, and proteolytic digestion with trypsin. The resulting peptides are quantified using multiple reaction monitoring mass spectrometry.^55^ For the genetic analysis, DNA is extracted from the DBS samples, enzymatically fragmented, and regions of interest are selectively enriched. The resulting DNA samples are analyzed using next-generation sequencing, multiplex ligation–dependent probe amplification, as well as whole genome sequencing in negative cases.^55^

### Emerging biomarkers and novel immunological assays

Biomarker research in HAE and other forms of angioedema is ongoing to address the unmet needs in diagnosis, family testing, disease activity monitoring, and assessment of the efficacy of therapeutics.^58^^,^^59^ In addition to the complement cascade biomarkers already used in the diagnosis of HAE (antigenic C1INH, fC1INH, antigenic C4), different biomarkers of the complement cascade (eg, protease-inhibitor complex C1INH–C1[r,s]), contact system and bradykinin-forming cascade (eg, bradykinin degradation products, cleaved high-molecular-weight kininogen [cHMWK], plasma kallikrein, activated blood clotting factor XII [FXIIa]), coagulation and fibrinolytic pathways, and endothelium-associated biomarkers have been proposed for diagnosis and monitoring of HAE (see Porebski et al, 2020, and Christiansen and Zuraw, 2024 for more details on emerging biomarkers in HAE).^59^^,^^60^ Potential biomarkers for HAE were also investigated in a metabolic analysis using high-performance liquid chromatography tandem mass spectrometry of urine samples from patients with HAE and healthy controls.^61^ In this analysis, 73 of 795 investigated metabolites, including those associated with purine metabolism, riboflavin metabolism, and tricarboxylic acid cycle, were significantly different in patients with HAE versus healthy controls.^61^ However, biomarkers identified in this study and other similar studies must be validated further, especially biomarkers involved in other clinical pathologies, biomarkers studied in small numbers of patients, and/or biomarkers with previously shown highly variable laboratory results.^59^^,^^61^

Validated biomarkers of kallikrein-kinin system activation could be helpful in differentiating mechanisms of angioedema.^62^ New methods to measure cHMWK, activated plasma kallikrein, and FXIIa are emerging as potential biochemical tests to identify bradykinin-mediated angioedema.^59^^,^^62^ Further to the standard fC1INH ELISA based on C1s protease inhibition, 2 ELISA assays for quantitation of fC1INH in HAE-C1INH diagnosis based on contact phase proteases have been described in the literature; one of these assays employs C1INH inhibition of plasma kallikrein, and the other one employs C1INH inhibition of FXIIa.^28^^,^^29^ These novel fC1INH ELISA assays were reported to have a similar performance compared with fC1INH chromogenic assay and traditional fC1INH ELISA assay based on C1s protease inhibition.^28^^,^^29^ An assay measuring fC1INH based on the residual enzymatic activity after contact phase activation using purified high-molecular-weight kininogen and plasma kallikrein mixed with FXIIa has also been described.^63^ The initial results have also been reported for 3 separate flow cytometry–based immunoassays using streptavidin-coated microbeads coated with biotinylated FXIIa, kallikrein, or C1s protease.^64^ Another study described the conversion of quantitative fC1INH immunoassay methods to a lateral flow assay platform as a proof-of-concept for a point-of-care assay to diagnose HAE.^65^

Inter-α-trypsin inhibitor heavy chain 4 (ITIH4) was also investigated as a novel biomarker for HAE diagnosis and monitoring, with 2 immunoassays for ITIH4 quantitation: one for intact, full-length ITIH4, and one for total ITIH4.^66^ The results of this study suggest that intact ITIH4 assay may be useful in HAE diagnosis, as intact ITIH4 was significantly lower in patients with HAE-C1INH compared with healthy controls.^66^ Furthermore, ITIH4 cleavage was suggested as a potential biomarker of HAE-nC1INH.^67^

## Digital tools for rare disease diagnosis

Ongoing research provides hope for technological advances to support diagnostic pathways in rare diseases, including HAE. Surveys on European data sources for machine learning approaches and interest in using machine learning for rare disease diagnosis suggest that, although there is a considerable interest in machine learning, there are also notable challenges.^68^^,^^69^ These challenges include lack of clinician experience, insufficient training, requirement to ensure the validity of data outputs, issues with the database quality, reservations from the database administrators about data use, and legislative challenges associated with using the data.^68^^,^^69^ The challenges in using database data to identify patients at risk for HAE can be illustrated by a study from Denmark, which did not find any digital features distinguishing between patients with known HAE and those with other types of phenotypically similar angioedema, even though this study only looked at the codes for previous diagnoses, procedures, treatments, operations, or examinations.^70^

The initial research to develop artificial intelligence models to aid in HAE diagnosis is underway. In one study, an artificial intelligence model was developed from US medical data (claims, prescriptions, and electronic medical records) and applied to a Japanese electronic medical record dataset.^71^ However, the sensitivity of this model in the Japanese dataset to correctly identify patients with known HAE was only 37.6%.^71^ This study also highlighted some challenges in developing artificial intelligence models for HAE diagnosis from medical records, including differences in coding between medical records from different countries, a small number of records from patients with HAE for machine learning due to the rarity of the disease, and challenges posed by incomplete medical records or medical records stored in databases of separate institutions.^71^ These digital tools may help to identify patients who may have HAE early in their diagnostic journey to aid in ensuring patients at risk are tested.

Another digital innovation for HAE diagnosis is an automated suspicion index screening tool currently in development, which uses electronic health record data to identify patients at risk for HAE.^72^ Furthermore, a proof-of-concept study for digital innovations in HAE investigated 3D photogrammetry facial imaging.^73^ The results of this study suggest that when there is facial involvement, facial imaging may improve patient outcomes by providing diagnostic assistance and disease monitoring.^73^

### Physician and patient awareness tools

In addition to the digital clinical suspicion tools that aim to raise suspicion based on electronic data, simple tools for identification of patients who raise a clinical suspicion of HAE in a clinical setting continue to be proposed. These include the HAAAAE (H4AE) warning signs,^74^ the Hereditary AngioEdema Rapid Triage (HAE-RT) tool,^75^ and the ABC warning signs for HAE.^76^ The H4AE signs include Hereditary, recurrent Angioedema, Abdominal pain, Absence of urticaria, Absence of response to antihistamines, and Estrogen association.^74^ The HAE-RT tool was proposed for emergency department use and contains 4 steps: (1) assess airway stability in patients with recurrent angioedema; (2) consider HAE in patients with no response to allergy treatments and past recurrent abdominal pain or swelling; (3) prompt treatment (with plasma-derived C1INH, icatibant, or, if others are not available, fresh frozen plasma); and (4) follow-up (referral to allergist to confirm diagnosis).^75^ The ABC warning signs for HAE diagnosis include A = angioedema, B = bradykinin, C = C1INH, D = distress factor, E = epinephrine nonresponsive, F = family history, and G = gastrointestinal and/or glottis edema.^76^ Tools promoting patient awareness have also been proposed; for example, the ACARE tool consists of a 10-item questionnaire related to HAE symptoms, treatments, and family disease history.^77^

In addition to the clinical history described above, imaging techniques may raise clinical suspicion of HAE and support in the early identification of HAE attacks as they occur. For example, abdominal and pelvic imaging using ultrasound or computed tomography may be helpful in identifying abdominal HAE attacks and in avoiding exploratory laparotomies.^78^

## Concluding remarks and future prospects

Countless patients with HAE worldwide continue to be undiagnosed or misdiagnosed and experience delays in obtaining the correct diagnosis of HAE, suggesting a persistent need for accessible and standardized diagnostic testing for HAE. Correct differential diagnosis and classification of different angioedema etiologies are also important. New developments in HAE diagnostics, such as tools to raise clinical suspicion based on patients’ electronic health records and DBS-based assays to aid wider access to diagnostic testing for HAE, may help support a simpler path to diagnosis for patients with HAE. Even with ongoing developments, physician education and increased awareness of HAE to promote the identification of patients who may need diagnostic testing will continue to be of utmost importance.

## Availability of data and sharing

Data sharing is not applicable to this article as no new data were created or analyzed.

## Authorship contribution

All authors contributed to the conceptulization and development of this manuscript and provided final approval for publication.

## Confirmation of unpublished work

The authors confirm that this manuscript is original, has not been published before, and is not currently being considered for publication elsewhere.

## Ethics approval

Not applicable.

## Consent for publication

All authors approved the final version and its submission.

## Funding

Takeda Development Center Americas, Inc. provided funding to Excel Scientific Solutions, Inc. for support in writing and editing this manuscript.

## Declaration of competing interest

ASG has received speaker/consultancy fees from Catalyst, CSL Behring, KalVista Pharmaceuticals, Multicare Pharma, Pharvaris, Pint Pharma, and Takeda; a scholarship from Brazilian Council of Research (CNPq); and a grant of researcher initiative from Takeda. MAR has received research support from BioCryst, BioMarin, CSL Behring, Ionis Pharmaceuticals, KalVista Pharmaceuticals, Pharvaris, and Takeda; has served as a consultant to Astria Therapeutics, BioCryst, BioMarin, CSL Behring, Cycle Pharma, Intellia Therapeutics, KalVista Pharmaceuticals, Ono Pharma, Pfizer, Pharming, Pharvaris, and Takeda; and provided speaker presentations for CSL Behring, Pharming, and Takeda. LC has received speaker fees from Takeda. SJ and DNE are employees of Takeda Development Center Americas, Inc. and hold stock/options in Takeda Pharmaceutical Company Limited. AZ has received speaker/consultancy fees from Astria Therapeutics, BioCryst, CSL Behring, KalVista Pharmaceuticals, Pharming, Pharvaris, and Takeda.
